# Correction: CD8 T Cell Response Maturation Defined by Anentropic Specificity and Repertoire Depth Correlates with SIVΔnef-induced Protection

**DOI:** 10.1371/journal.ppat.1004915

**Published:** 2015-05-14

**Authors:** The PLOS Pathogens Staff

## Notice of Republication

This article was republished on April 15, 2015 to correct an error where the Table 1 legend was included in the main text of the article. The publisher apologizes for these errors, which were introduced during the typesetting process. Please download this article again to view the correct version. The originally published, uncorrected article and the republished, corrected article are provided here for reference.

## Supporting Information

S1 File Originally published, uncorrected article. (PDF)

S2 File Republished, corrected article. (PDF)
