# Supplementary material for: CD8 T Cell Response Maturation Defined by Anentropic Specificity and Repertoire Depth Correlates with SIVΔnef-induced Protection
Source: PLoS Pathog. 2015 Feb 17;11(2):e1004633. doi: 10.1371/journal.ppat.1004633 (PMC4334552; doi:10.1371/journal.ppat.1004633)
Supplement: S1 Table — The mapped CD8 T cell response in peripheral blood (PBMC, left) and lymph nodes (LN, right) for animal 225–97 at week 40 and animal 211–02 at week 20 post-SIVΔnef vaccination. The frequency of an epitope-specific response per 106 cells and the epitope sequence are listed. Concordant CD8 T cell responses between peripheral blood and lymph nodes are shaded in green. (DOCX) [file ppat.1004633.s001.docx]

| **225-97** |  |  |  |  |  |
| --- | --- | --- | --- | --- | --- |
| **PBMC** |  |  | **LN** |  |  |
| **Name** | **Freq/10^6^ Cells** | **Sequence** | **Name** | **Freq/10^6^ Cells** | **Sequence** |
| Env 28 | 1135 | CNKSETDRWGL | Env 28 | 416 | CNKSETDRWGL |
| Env 84 | 355 | QPINDRPKQAW | Env 158 | 216 | TPKWNNETWQEWERK |
| Env 158 | 595 | TPKWNNETWQEWERK | Env 215 | 432 | RRGGRWILAIP |
| Env 203 | 670 | LQRIREVLRTELTYL | Pol 73 | 168 | NRVTQDFTEVQLGIP |
| Env 215 | 1100 | RRGGRWILAIP | Pol 124 | 144 | LTEEVQWTEMA |
| Pol 124 | 340 | LTEEVQWTEMA | Tat 8 | 128 | TPESANLGEEILSQL |
| Tat 8 | 490 | TPESANLGEEILSQL | Vif 13 | 112 | WAWWTCSRVIFPLQE |
|  |  |  |  |  |  |
|  |  |  |  |  |  |
|  |  |  |  |  |  |
| **211-02** |  |  |  |  |  |
| **PBMC** |  |  | **LN** |  |  |
| **Name** | **Freq/10^6^ Cells** | **Sequence** | **Name** | **Freq/10^6^ Cells** | **Sequence** |
| Env 84 | 335 | QPINDRPKQAW | Env 84 | 132 | QPINDRPKQAW |
| Gag 68 | 500 | LGLQKCVRMYNPTNI | Gag 64 | 120 | QQNPIPVGNIY |
| Gag 98 | 265 | KPIKCWNCGKEGHSA | Gag 68 | 360 | LGLQKCVRMYNPTNI |
| Gag 118 | 290 | YMQLGKQQREK | Nef 12 | 288 | GLDKGLSSLSC |
| Nef 12 | 395 | GLDKGLSSLSC | Tat 8 | 204 | TPESANLGEEILSQL |
| Tat 8 | 300 | TPESANLGEEILSQL | Tat 11 | 120 | SQLYRPLEACY |

**Supplementary Table 1: Epitope mapping of the CD8 T cell response in peripheral blood and lymph nodes.**

The mapped CD8 T cell response in peripheral blood (PBMC, left) and lymph nodes (LN, right) for animal 225-97 at week 40 and animal 211-02 at week 20 post-SIVΔnef vaccination. The frequency of an epitope-specific response per 10^6^ cells and the epitope sequence are listed. Concordant CD8 T cell responses between peripheral blood and lymph nodes are shaded in green.
