# Supplementary material for: Correction: CD8 T Cell Response Maturation Defined by Anentropic Specificity and Repertoire Depth Correlates with SIVΔnef-induced Protection
Source: PLoS Pathog. 2015 May 14;11(5):e1004915. doi: 10.1371/journal.ppat.1004915 (PMC4431722; doi:10.1371/journal.ppat.1004915)
Supplement: S2 File — (PDF) [file ppat.1004915.s002.pdf]

PLOS | PATHOGENS

RESEARCH ARTICLE

CD8 T Cell Response Maturation Defined by Anentropic Specificity and Repertoire Depth Correlates with SIVΔnef-induced Protection

Sama Adnan[1], Arnaud D. Colantonio[1], Yi Yu[1], Jacqueline Gillis[1], Fay E. Wong[1], Ericka A. Becker[2], Michael Piatak Jr[3†], R. Keith Reeves[1,4], Jeffrey D. Lifson[3], Shelby L. O'Connor[2], R. Paul Johnson[1,5,6]*

1 New England Primate Research Center, Harvard Medical School, Southborough Campus, Southborough, Massachusetts, United States of America, **2** Wisconsin National Primate Research Center, University of Wisconsin, Madison, Wisconsin, United States of America, **3** AIDS and Cancer Virus Program, Leidos Biomedical Research, Inc., Frederick National Laboratory, Frederick, Maryland, United States of America, **4** Center for Virology and Vaccine Research, Beth Israel Deaconess Medical Center, Boston, Massachusetts, United States of America, **5** Ragon Institute of MGH, MIT and Harvard, Cambridge, Massachusetts, United States of America, **6** Yerkes National Primate Research Center, Emory University, Atlanta, Georgia, United States of America

† Deceased
* pjohnson@hms.harvard.edu, paul_johnson@hms.harvard.edu

Abstract

The live attenuated simian immunodeficiency virus (LASIV) vaccine SIVΔnef is one of the most effective vaccines in inducing protection against wild-type lentiviral challenge, yet little is known about the mechanisms underlying its remarkable protective efficacy. Here, we exploit deep sequencing technology and comprehensive CD8 T cell epitope mapping to deconstruct the CD8 T cell response, to identify the regions of immune pressure and viral escape, and to delineate the effect of epitope escape on the evolution of the CD8 T cell response in SIVΔnef-vaccinated animals. We demonstrate that the initial CD8 T cell response in the acute phase of SIVΔnef infection is mounted predominantly against more variable epitopes, followed by widespread sequence evolution and viral escape. Furthermore, we show that epitope escape expands the CD8 T cell repertoire that targets highly conserved epitopes, defined as anentropic specificity, and generates de novo responses to the escaped epitope variants during the vaccination period. These results correlate SIVΔnef-induced protection with expanded anentropic specificity and increased response depth. Importantly, these findings render SIVΔnef, long the gold standard in HIV/SIV vaccine research, as a proof-of-concept vaccine that highlights the significance of the twin principles of anentropic specificity and repertoire depth in successful vaccine design.

Author Summary

Annually, more than two million people are infected with HIV, the virus that causes AIDS. Due to the ability of the virus to escape host immune responses, designing a

OPEN ACCESS

Citation: Adnan S, Colantonio AD, Yu Y, Gillis J, Wong FE, Becker EA, et al. (2015) CD8 T Cell Response Maturation Defined by Anentropic Specificity and Repertoire Depth Correlates with SIVΔnef-induced Protection. PLoS Pathog 11(2): e1004633. doi:10.1371/journal.ppat.1004633

Editor: Ronald Swanstrom, University of North Carolina at Chapel Hill, UNITED STATES

Received: August 5, 2014

Accepted: December 16, 2014

Published: February 17, 2015

Copyright: This is an open access article, free of all copyright, and may be freely reproduced, distributed, transmitted, modified, built upon, or otherwise used by anyone for any lawful purpose. The work is made available under the Creative Commons CC0 public domain dedication.

Data Availability Statement: All relevant data are within the paper and its Supporting Information files.

Funding: This was work was funded by the National Institutes of Health, NIAID grants AI071306, AI095985, as well as grant P51 OD011103 (formerly RR00168) from the Office of Research Infrastructure Programs. SA is supported by the Harvard Multidisciplinary Training grant (T32 AI07387-24). The funders had no role in study design, data collection and analysis, decision to publish, or preparation of the manuscript.

Competing Interests: The authors have declared that no competing interests exist.

successful HIV vaccine has been elusive. Similar to HIV in humans, rhesus macaques can be infected with SIV, a close relative and ancestor of HIV, resulting in simian AIDS. SIVΔnef, a live attenuated form of SIV, protects rhesus macaques from subsequent challenge with pathogenic SIV and is widely viewed as the most effective SIV vaccine. Here, we demonstrate that after vaccination of macaques with SIVΔnef, the immune response initially targets more variable regions of the virus, which the virus rapidly escapes. However, as the virus escapes, the immune response evolves to target more conserved regions of the virus as well as escape variants. This refocused targeting of conserved regions by the immune response provides a new mechanistic model that contributes to our understanding of how SIVΔnef vaccination protects animals from pathogenic challenge with SIV. Our findings also reinforce the importance of developing HIV vaccines that target conserved regions of the virus as well as their potential variants.

Introduction

Two decades ago, a report described a cohort of rhesus macaques infected with the live attenuated simian immunodeficiency virus (LASIV), SIVΔnef, and subsequently protected from pathogenic wild-type SIV challenge [1]. Since then, numerous studies have demonstrated the efficacy of SIVΔnef-induced protection, ranging from complete protection with sterilizing immunity to partial protection with two or more logs reduction in peak and set-point viremia [2–6]. Remarkably, presumably in part due to the replication of SIVΔnef in mucosal sites [7], robust protection has been also been documented following mucosal challenges [3–5]. SIVΔnef has also induced significant protection against heterologous challenge, albeit less effectively than against homologous challenge [4,5]. However, studies describing disease progression in SIVΔnef-vaccinated infant macaques and a subset of adult non-human primates precluded live attenuated HIV from being developed as a vaccine in human subjects [8–10].

Due to concerns over safety, research on SIVΔnef and related LASIV vaccines has shifted from safety-and-efficacy determination to mechanism-of-action delineation. As the most effective lentiviral vaccine, SIVΔnef has been extensively studied in order to shed light on the correlates of vaccine-mediated protection. However, no immunological correlate or mode of action has consistently been identified as being responsible for protection against pathogenic challenge. SIVΔnef generates a diverse SIV-specific antibody response [11,12] and macaques vaccinated with the related attenuated virus SIVΔ3 and lacking the *Mamu-A∗01* MHC I allele show effective control of pathogenic viral challenge despite CD8 T cell depletion [13], implying that humoral immunity may play a significant role, at least in some genotypic backgrounds. Innate immunity has also been implicated as a correlate of SIVΔnef-induced protection [3]. SIVΔnef infection induces potent CD8 T cell responses similar in magnitude to wild-type SIV infection [14], and multiple studies have implicated SIV-specific CD8 T cells in the protection induced by SIVΔnef [4–6,15]. Most recently, Fukazawa et al. [6] correlated the magnitude of lymph node SIV-specific T cell responses with protection elicited by a range of different LASIV strains, including SIVΔnef. Finally, studies in a related vaccine challenge model involving vaccination with an attenuated SHIV strain have implicated SIV-specific CD8 T cell responses in the female reproductive tract as playing a crucial role in protection [16].

In this work, we sought to scrutinize the interplay of the attenuated virus and the immune response at a high resolution. We chose to focus on the CD8 T cell response as the most likely immune correlate for protection given that previous studies have demonstrated: potent antilentiviral activity of CD8 T cells in vitro [17–21]; increased viremia during chronic SIV

infection after CD8 T cell depletion [22]; the ability of a live attenuated SHIV vaccine containing HIV *env* to protect vaccinated macaques against SIVmac239 challenge [16,23]; SIVΔnef-mediated protection of animals challenged with wild-type SIVmac239 containing highly heterologous *env* sequences [24]; and a correlation between increased LASIV-induced protection and higher magnitude of SIV-specific CD4 and CD8 T cells in the lymph nodes of vaccinated animals [6].

A distinguishing feature of the SIVΔnef and SIVΔ3 vaccines is the gradual increase in protection against wild-type SIV challenge during the first 15–20 weeks of vaccination [2,25]. Given the low-level replication rate of SIVΔnef after initial control of viremia by 8–12 weeks after vaccination, it was unknown whether SIVΔnef undergoes sequence evolution after acute infection. The only previously documented sequence evolution of SIVΔnef was in the Tat SL8 epitope, which escaped as early as 3 weeks post-infection [26]. We hypothesized that, despite low levels of viral replication, SIVΔnef undergoes sequence evolution during the vaccination period, which would induce a shift in the specificity of the CD8 T cell response between week 5, when the animals are not protected, and week 20, where increased protection plateaus [12]. We made use of high-throughput deep sequencing to quantify SIVΔnef sequence variants and their frequencies in vaccinated animals, including those with undetectable plasma viral loads. Concurrently, we fine-mapped the CD8 T cell responses at the epitope level at week 5 and 20 post-vaccination. Studying this cohort of animals longitudinally after SIVΔnef vaccination, we demonstrate concomitant coevolution of the virus and the specificity of the CD8 T cell response, resulting in a CD8 T cell repertoire with increased anentropic specificity, wider cumulative breadth, rearranged immunodominance and enhanced depth.

Materials and Methods

Ethics statement

The animals included in this study were all female Indian-origin rhesus macaques (*Macaca mulatta*), housed in a biocontainment facility at the New England Primate Research Center (NEPRC). These experiments and procedures were approved by the Harvard Medical Area Standing Committee on Animals. At approval of the study, the assigned protocol number by the Institutional Animal Care and Use Committee (IACUC) was 04383. The Harvard Medical School animal management program is accredited by the Association for the Assessment and Accreditation of Laboratory Animal Care, International (AAALAC), and meets National Institutes of Health standards as set forth in the 8th edition of the Guide for the Care and Use of Laboratory Animals [27]. The institution also accepts as mandatory the PHS Policy on Humane Care and Use of Laboratory Animals by Awardee Institutions and NIH Principles for the Utilization and Care of Vertebrate Animals Used in Testing, Research, and Training. There is on file with the Office of Laboratory Animal Welfare (OLAW) an approved Assurance of Compliance (A3431-01).

All animals were housed indoors in an SOP-driven, AAALAC-accredited facility. Husbandry and care met the guidance of the Animal Welfare Regulations, OLAW reporting and the standards set forth in The Guide for the Care and Use of Laboratory Animals. All research animals were enrolled in the NEPRC behavioral management program, including an IACUC-approved plan for Environmental Enrichment for research primates. This program included regular behavioral assessments, and provision of species appropriate manipulanda, and foraging opportunities. This protocol had an IACUC-approved exemption from social housing based on scientific justification. Primary enclosures consisted of stainless steel primate caging provided by a commercial vendor. Animal body weights and cage dimensions were regularly monitored. Overall dimensions of primary enclosures (floor area and height) met the

specifications of The Guide for the Care and Use of Laboratory Animals, and the Animal Welfare Regulations (AWR's). Further, all primary enclosures were sanitized every 14 days at a minimum, in compliance with AWRs. Secondary enclosures (room level) met specifications of The Guide with respect to temperature, humidity, lighting and noise level. The animals were provided ad lib access to municipal source water, offered commercial monkey chow twice daily, and offered fresh produce a minimum of three times weekly. Light cycle was controlled at 12/12 hours daily. The animals were subject to twice daily documented observations by trained animal care and veterinary staff, and enrolled in the facility's environmental enrichment, and preventative health care programs. Euthanasia took place at defined experimental endpoints using protocols consistent with the American Veterinary Medical Association (AVMA) guidelines. Animals were first sedated with intramuscular ketamine hydrochloride at 20 mg/kg body followed by sodium pentobarbital (≥100 mg/kg) intravenously to achieve euthanasia.

Peptide pools

To determine SIV protein-specific T cell responses, 9 whole protein peptide pools were constructed containing 15-mer peptides overlapping by 11 amino acids at 2.5 μg/ml for each. Peptides spanning the SIVmac239 proteome and corresponding to the peptide sequences available from the AIDS Research and Reference Reagent Program, Division of AIDS, NIAID, NIH, were synthesized by the Massachusetts General Hospital core peptide facility. The sequence of individual peptides can be found at www.aidsreagent.org.

For epitope mapping, a three-dimensional peptide matrix was constructed. The matrix was composed of 87 peptide pools, each containing around 27 to 30 peptides. The 87 peptide pools encompassed the entire SIV proteome with a coverage of 3-fold, such that each of the 823 overlapping peptides spanning the SIVmac239 proteome was represented in 3 different pools. The peptides used for epitope mapping were 15-mer peptides overlapping by 11 amino acids and were obtained from the AIDS Research and Reference Reagent Program, Division of AIDS, NIAID, NIH. The sequence of individual peptides can be found at www.aidsreagent.org.

Calculation of entropy

The entropy for each epitope within SIVmac239 was determined by calculating the entropy of each 9-amino-acid window within SIVmac239, which was determined by aligning 10 proteomes of SIVsmm, the ancestral virus of both the clonal SIVmac239 and the quasi-species SIVmac251. The aligned proteome sequences were then uploaded into the Shannon Entropy-One calculator from the Los Alamos National Database (http://www.hiv.lanl.gov/content/sequence/ENTROPY/entropy_one.html). The program calculates an entropy score for each 9 amino acid sequence in the SIVsmm proteome.

CD4 T cell separation

In order to detect only IFN-γ-producing CD8 T cells, ELISPOT assays were carried out with PBMCs or lymph node mononuclear cells (LNMC) after negative selection of CD4 T-lymphocyte populations fractionated by magnetic bead separation (CD4 Dynabeads; Dynal, Oslo, Norway) as previously described [20]. Negatively selected CD4 T cells were >90% CD8 T cells. CD4 T cell-depleted PBMC and LNMC were suspended in R-10 medium and used the same day in ELISPOT assays. Isolated lymph node CD4 T cells were pelleted and used for RNA extraction and cell-associated viral RNA quantitation and sequencing.

Determination of plasma viral loads

For quantitation of plasma viral loads in SIVΔnef-infected animals, highly specific, real-time RT-PCR assays were performed as described previously [28]. The assay specific for SIVmac239Δnef was developed by designing a reverse primer that uniquely recognizes the sequence generated by the deletion of *nef* coding sequences in SIVmac239. The nominal threshold for this assay was 30 viral RNA copy equivalents/ml plasma.

Sequencing of SIVΔnef

To sequence lymph node cell-associated viral RNA, cell-associated viral loads were first determined, incorporating qPCR assay primers and probes for SIV *gag*, rhesus *CCR5* and SIVmac239Δnef as described [28]. Briefly, pelleted cells were rapidly disrupted in 100 μl lysis/digestion solution (3M GuHCl, 50 mM TrisCl, pH 7.6, 1 mM $CaCl_2$ and 1 mg/ml Proteinase K), utilizing a Branson 450 sonifier equipped with a high-intensity cup horn and set at 60% power amplitude (Branson Ultrasonics, Danbury, CT). After continued digestion at 42°C for 1 hour, 400 μl of GuSCN/carrier solution (~5.7 M GuSCN, 50 mM TrisCl, pH 7.6, 1 mM EDTA, 600 μg/ml glycogen) was added to completely dissociate RNA-protein complexes. Total nucleic acids were precipitated by addition of 500 μl isopropanol, collected by centrifugation, and washed with 70% ethanol. This precipitate was air-dried, suspended in 60 μl 1X TurboDNAse buffer (ThermoFisher/Ambion), and split into 2 equal 30 μl aliquots for separate RNA and DNA preparation. The samples for DNA determinations were denatured by heating to 100°C for 5 min and quenched on ice prior to qPCR. Associated with determinations of SIVmac239Δnef DNA, cell equivalents based on qPCR for rhesus CCR5 at single haploid copy per genome (2 copy equivalents per cell) were determined on aliquots diluted 1:10 with 5 mM TrisCl, pH 9.0. Copy equivalents of SIVgag and SIVmac239Δnef targets were determined as described [28]. For the aliquots reserved for RNA, 20 μl of a cocktail containing 2 μl (4 Units) TurboDNAseI in 1X buffer (ThermoFisher/Ambion) were added to each, and the samples incubated at 42°C for 30 min to digest DNA. After digestion, RNA was recovered by addition of 200 μl of GuSCN solution without glycogen carrier, followed by precipitation with 250 μl isopropanol and a 70% ethanol wash, as noted above. Genome equivalents based on SIV *gag* or SIVmac239Δnef target sequences were determined as described. The threshold limits of detection for both SIV DNA and SIV RNA were 30 total copies of SIV sequence normalized to calculated cell equivalents.

Plasma samples were spun at 14,000 rpm at 4°C for 1 hour in a microcentrifuge. Viral RNA was then isolated using the Qiamp MinElute Virus spin kit according to manufacturer's instructions. Plasma viral RNA and cell-associated viral RNA (isolated and quantitated as described above) were reverse transcribed and amplified using 4 sets of primers to produce 4 overlapping amplicons spanning the entire length of the viral coding sequence using the SuperScript III High Fidelity One-Step RT-PCR kit (Invitrogen, Life Technologies, Carlsbad, CA). PCR products were either purified with the Qiagen MinElute Gel Extraction (Qiagen) kit or Agencourt AMPure XP beads (Beckman Coulter). Samples were quantified with the Quant-IT dsDNA HS Assay kit (Invitrogen)

The four resultant amplicons were combined and then libraries were created and tagged using the Nextera DNA Sample Prep Kit (Illumina). Individually tagged libraries were quantified with the Quant-IT dsDNA HS Assay kit (Invitrogen) and the Agilent High Sensitivity DNA kit (Agilent Technologies). Libraries were then pooled together, denatured with NaOH, and run on an Illumina MiSeq using either a 300 or 500 cycle MiSeq Kit (Illumina, San Diego, CA, USA).

IFN-γ ELISPOT assays

IFN-γ-producing T-cell responses were enumerated using an enzyme-linked immunospot (ELISPOT) assay for detection of macaque IFN-γ (Mabtech, Mariemont, OH). CD4-depleted PBMC and LN mononuclear cells were stimulated at $2x10^5$ cells per well with peptide pools (15-mer peptides overlapping by 11 amino acids at 2.5 μg/ml each). Cells were incubated overnight in multiscreen plates (Millipore) coated with an IFN-γ capture antibody, and spots representing IFN-γ-producing T cells were detected in an enzyme-linked, colorimetric assay for bound IFN-γ. Spots were counted using an automated ELISPOT plate reader (Zellnet Consulting, New York, NY).

For epitope mapping, peptide-pool-stimulated wells, containing a number of spots 3 times higher than background and greater than 30 spots per million mononuclear cells, were selected for second round deconvolution. To deconvolute peptide-specific CD8 T cell responses, peripheral and lymph node mononuclear cells, depleted of CD4 T cells, were stimulated with single peptides that were present in at least 2 positive pools of the 87 peptide pools. Wells with responses to single peptides 3 times higher than background and greater than 60 spots per million were considered significant.

Typing of MHC I alleles

Comprehensive MHC I typing using pyrosequencing was conducted as described [29]. Total cellular RNAs were converted to cDNA using a Superscript III First-Strand Synthesis System (Invitrogen). Primary cDNA-PCR amplicons spanning 190 bp of exon two of macaque class I sequences were generated with high-fidelity Phusion polymerase (New England Biolabs). Each PCR primer contained one of 12 distinct 10 bp MID tags along with adaptor sequences for 454 pyrosequencing. After purification, primary amplicons were normalized to equimolar concentrations and groups of 12 animals were pooled for GS FLX analysis. The emulsion PCR and pyrosequencing steps were performed with Genome Sequencer FLX instruments (Roche/454 Life Sciences) using GS FLX protocols according to the manufacturer's specifications (454 Life Sciences) at the 454 Sequencing Center (Branford, CT) and the University of Illinois at Urbana-Champaign High-Throughput Sequencing Center.

Polychromatic flow cytometry analyses and tetramer staining

Surface staining was carried out by standard procedures for our laboratory as described [30]. Except where noted, all reagents were obtained from BD Biosciences (San Diego, CA) and included monoclonal antibodies to the following molecules: CD3 (clone SP34-2, APC-Cy7 conjugate) CD4 (clone SK3, PerCP-Cy5.5 conjugate), CD8α (clone RPA-T8, Alexa700 conjugate), CD28 (clone CD28.2, PE-Texas Red conjugate, Beckman-Coulter, Fullerton, CA), CCR7 (clone 150503, Pacific Blue conjugate, custom), KI-67 (clone EH12.2H7, PE conjugate, custom), CD127 (clone R34.34, PE conjugate, Beckman-Coulter), perforin (clone Pf-344, FITC conjugate, Mabtech, Mariemont, OH). Intracellular staining for perforin and KI-67 expression was performed using Caltag Fix & Perm (Invitrogen, Camarillo, CA) according to the manufacturer's suggested protocol. Enumeration of SIV-specific cells using PE- or APC-conjugated tetramers to Mamu-A∗01 $Gag_{181-189}$CM9 and Tat_{28-35}SL8 (kindly provided by Nancy Wilson and David Watkins, Wisconsin National Primate Research Center, Madison Wisconsin) was performed as described previously [31]. All acquisitions were made on an LSR II (BD Biosciences) and analyses were done using FlowJo software (Tree Star Inc., Ashland, OR). Isotype-matched controls and/or fluorescence-minus-one (FMO) controls were included in all assays [32].

Statistical analysis

All statistical analyses were done using GraphPad Prism software (GraphPad Software v6.0b, Inc., La Jolla, CA, USA). Non-parametric Wilcoxon and Mann–Whitney tests were used for statistical analysis where the sample size was less than 6. Otherwise, parametric t tests were conducted; p values less than 0.05 were assumed to be significant in all analyses.

Results

Widespread sequence evolution occurs after SIVΔnef infection

To study the effect of epitope escape on the evolution of the CD8 T cell response following vaccination with SIVΔnef, we first assessed the extent of viral sequence variation in 12 animals after SIVΔnef infection using next generation sequencing (NGS). The vaccinated animals, most of which displayed classical SIVΔnef viral replication kinetics of peak viremia at week 2 and viral set-points at or near undetectable levels (<30 copy Eq/ml) at week 8, were followed for 20 to 40 weeks before challenge (Fig. 1A). Plasma viral samples from the 12 vaccinated animals were sequenced at time points ranging from 1 week to 34 weeks post-vaccination.

RT-PCR was used to create four overlapping amplicons corresponding to the full-length SIV viral genome. Libraries were prepared from the pool of four amplicons and then sequenced using the Illumina MiSeq platform. Although there was already widespread sequence variation during the first 2 weeks after vaccination with SIVΔnef, none of the variants in any of the animals sequenced exceeded a threshold of 10% (Figs. 1C and S1), suggesting that these variants were not subject to significant selection in vivo during this initial observation period. However, by week 4 post-vaccination, as the viral load declined from peak viremia, there was a significant increase in the prevalence of nonsynonymous mutations that exceeded the 10% threshold. At this time point, plasma viral RNA samples from 12 animals displayed an average of 8 nonsynonymous mutations, each with a greater frequency than 10% (Figs. 1C and S2). To determine if sequence variation continued to increase between weeks 4 and 20, plasma viral RNA was sequenced from the 3 macaques with detectable viremia at week 20. Plasma viral RNA samples from these 3 animals at week 20 had an average of 28 nonsynonymous mutations with a frequency greater than 10%, a marked increase compared to an average of 8 nonsynonymous mutations at week 4 (Figs. 1C and S3).

To determine the extent of sequence evolution in SIVΔnef-vaccinated animals that had lower levels of plasma viremia, we analyzed lymph node CD4 T cell-associated viral RNA at week 20 from 3 animals. Follicular helper CD4 T cells, which are localized in secondary lymphoid tissues, are preferentially infected by SIVΔnef [33], and therefore have higher cell-associated viral RNA levels than CD4 T cells in peripheral blood [6]. Sequencing of lymph node CD4 T cell-associated viral RNA at week 20 revealed increased viral sequence variation even in SIVΔnef-infected animals with undetectable plasma viremia, albeit at lower levels of variation than observed for viremic animals. Lymph node associated viral RNA sequenced from animals with undetectable plasma viremia had an average of 15 nonsynonymous mutations with a greater frequency than 10% (Fig. 1D), a substantial increase over the 8 nonsynonymous mutations at week 4 for these animals (S2 Fig.).

SIVΔnef was longitudinally sequenced in 5 animals, from plasma virus at week 4 and either plasma virus or LN CD4 T cell-associated virus at week 20. Pooling all the variants (greater than 10% frequency) for all 5 animals, the average frequency of nonsynonymous mutations increased from 29% at week 4 to 47% at week 20 ($p = 0.001$) (Fig. 1B). Moreover, there was a significant accumulation in the number of nonsynonymous mutations, increasing from an average of 10 nonsynonymous mutations per animal at week 4 to an average of 24 at week 20

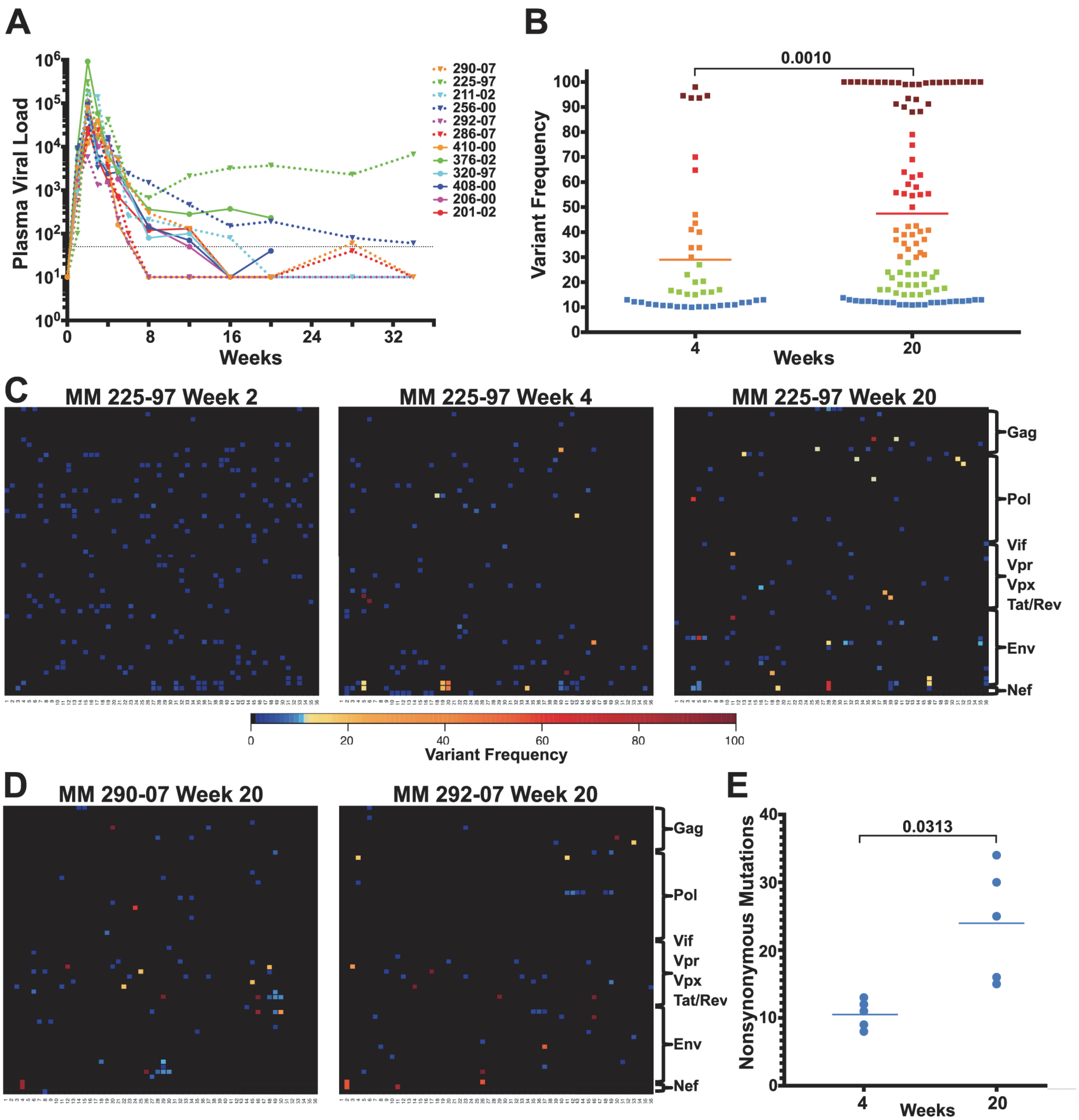

Fig 1. Extensive sequence evolution in SIVΔnef. (A) Plasma viral loads (RNA copies/ml) of 12 SIVΔnef-infected animals. The dashed black line marks the 30 copies/ml threshold. (B) Frequency of nonsynonymous mutations (>10% variant frequency) increased significantly ($p = 0.001$) between weeks 4 and 20 for the 5 animals whose virus was sequenced at both time points. Squares are colored according to variant frequency: blue: 10–15%, green: 16–30%, orange: 31–50%, red: 51–80%, and dark red: 81–100%. (C) The sequence evolution in animal 225–97 at weeks 2, 4 and 20 is displayed for every amino acid in the SIVΔnef proteome. The top left corner is the first amino acid of Gag and the bottom right corner is the last expressed amino acid of the truncated Nef. (D) Extensive SIVΔnef sequence evolution in animals 290–07 and 292–07 at week 20, despite undetectable plasma viral load. Lymph node cell-associated viral RNA was isolated for sequencing. (E) Significant increase ($p = 0.031$) in the number of nonsynonymous mutations between weeks 4 and 20 for 5 animals whose virus was sequenced at both time points.

doi:10.1371/journal.ppat.1004633.g001

(p = 0.031) (Fig. 1E). In all animals whose virus was sequenced, there was extensive sequence evolution as measured by the number of nonsynonymous mutations and their frequencies, including in animals with undetectable plasma viral loads.

Evolution of the specificity of the CD8 T cell response in SIVΔnef-vaccinated animals

To determine if viral sequence evolution was accompanied by a shift in the epitopes targeted by the SIV-specific CD8 T cell response, we analyzed CD8 T cell responses at weeks 5 and 20 in the 12 vaccinated animals using IFN-γ ELISPOT assays using overlapping peptide pools spanning the entire SIV proteome. In contrast to increased protection between weeks 5 and 20, there was a 40% decrease in the magnitude of the SIV-specific CD8 T cell response in peripheral blood mononuclear cells (PBMC) between weeks 5 and 20 (Fig. 2A). Similarly, the breadth of the CD8 T cell response did not correlate with increased protection between weeks 5 and 20, as the number of positive pools remained stable, with an average of 6 stimulating pools detected at week 5 and an average of 5.2 pools detected at week 20 in PBMCs (Fig. 2B).

A recent report demonstrated that the magnitude of the SIV-specific CD8 T cell response in lymph nodes at week 50 post-vaccination with SIVΔnef correlated with protection against

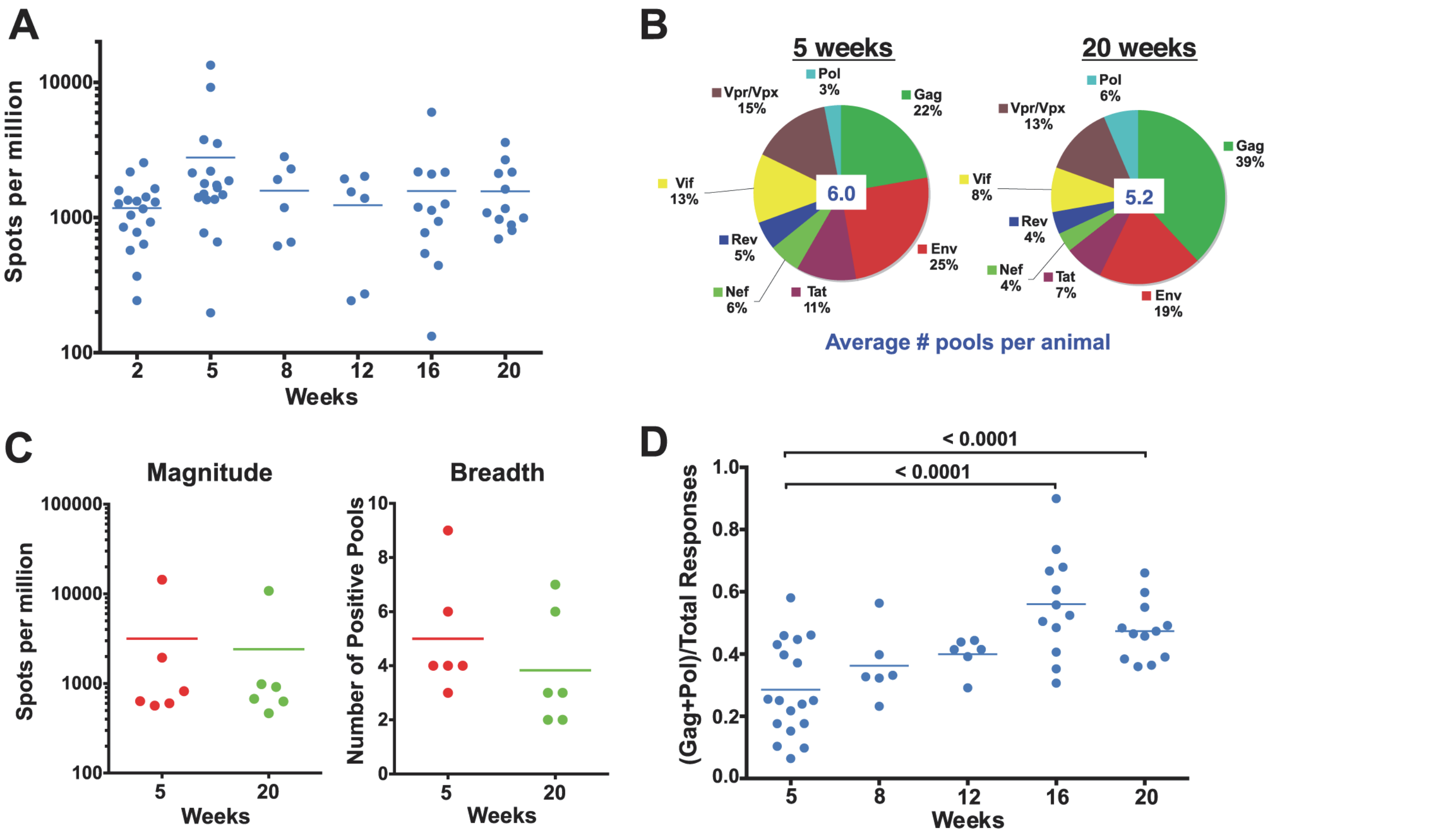

Fig 2. Evolution of the epitope specificity of SIV-specific CD8 T cell responses in SIVΔnef-vaccinated animals. (A) The total magnitude of the PBMC CD8 T cell response as measured by IFN-γ ELISPOT assays using whole protein overlapping peptide pools. (B) The breadth of the PBMC CD8 T cell response as measured by the average number of positive pools at weeks 5 and 20. (C) The magnitude and the breadth of lymph node CD8 T cell responses. The total magnitude of lymph node CD8 T cell response to SIV was determined by adding the responses for all 30 peptide pools spanning the entire SIVΔnef proteome. (D) The Gag and Pol-specific CD8 T cell response as a fraction of the total SIV-specific CD8 T cell response increased significantly between weeks 5 and 16/20 ($p<0.0001$), plateauing at week 16.

doi:10.1371/journal.ppat.1004633.g002

intravenous challenge [6]. To determine if the kinetics of the SIV-specific CD8 T cell response in lymph nodes (LN) correlated with the increased protection observed between weeks 5 and 20, we looked at the magnitude and the breadth of the SIV-specific CD8+ T cell response in lymph nodes. Similar to the magnitude of the CD8 T cell response observed in peripheral blood, the magnitude of the SIV-specific CD8 T cell response in lymph nodes decreased modestly, by 25%, between weeks 5 and 20 (Fig. 2C). The breadth of the response, as defined by recognition of peptide pools of 30 peptides each, in the lymph nodes was likewise comparable to the breadth of the response in the periphery (Fig. 2C), with a slight decrease from 5 positive pools at week 5 to 4 positive pools at week 20.

We next examined CD8 T cell responses to Gag and Pol, the two most conserved proteins in lentiviruses, as a fraction of total responses to the SIV proteome, an indicator of the level of conservation of the SIV-specific CD8 T cell response. Intriguingly, there was a significant enrichment of the ratio of the magnitude of Gag and Pol responses to the total SIV-specific CD8 T cell response between weeks 5 and 20 in SIVΔnef-vaccinated animals ($p = <0.0001$) (Fig. 2D). The ratio of Gag and Pol responses to total SIV responses increased in every animal between week 5 and week 20, increasing from an average of 28% at week 5 to 47% at week 20.

Epitope mapping reveals a change in the CD8 T cell repertoire and immunodominance

To characterize the shift in CD8 T cell specificity at the level of individual epitopes, we mapped the CD8 T cell response to single peptides for 6 animals at weeks 5 and 20 post-vaccination with SIVΔnef. Epitope mapping of SIV-specific CD8 T cell responses was conducted on CD8 T cells from lymph nodes. For the 2 animals whose SIV-specific CD8 T cell responses were mapped in both lymph nodes and peripheral blood, there was no significant difference in the epitopes targeted by the CD8 T cell responses in these two sites (S1 Table).

Longitudinal epitope mapping in SIVΔnef-vaccinated animals revealed a change in the relative immunodominance of recognized epitopes between weeks 5 and 20 after SIVΔnef vaccination. Furthermore, the specific SIV epitopes recognized by CD8 T cell responses differed significantly between week 5 and week 20. Of the 36 CD8 T cell epitopes mapped in the 6 animals at week 20, only 14 were also recognized at week 5 (Figs. 3A and S4). Almost two thirds of the responses at week 20 are de novo responses or were not readily detectible at week 5.

As a first step in assessing whether these shifts in epitope specificity reflected increased anentropic specificity of the CD8 T cell response, we examined the conservation of targeted epitopes at different times after SIVΔnef vaccination. We assigned every mapped epitope a conservation score by calculating the mean entropy of nine amino acid stretches for all SIV proteins, as determined from the alignment of 10 SIVsmm predicted open reading frames (Fig. 4). SIVsmm strains were chosen because the virus clone SIVmac239 and the quasispecies SIVmac251 are derived from SIVsmm. Interestingly, in 5 out of 6 animals, the immunodominant response targeted a more conserved epitope (lower entropy score) at week 20 than at week 5 ($p = 0.039$) (Fig. 3B). Moreover, 7 of the de novo CD8 T cell responses detected at week 20 targeted highly conserved epitopes with an entropy lower than 0.1.

Epitope escape drives CD8 T cell anentropic specificity

Having established the accumulation of sequence variants between weeks 5 and 20 and a simultaneous shift in the SIV epitopes targeted by the CD8 T cell response, we sought to determine whether the two events were causally related. Overlaying epitopes mapped onto the sequenced virus from the 6 animals vaccinated with SIVΔnef demonstrated that there was a high concordance between the occurrence of nonsynonymous mutations and identification of CD8 T cell

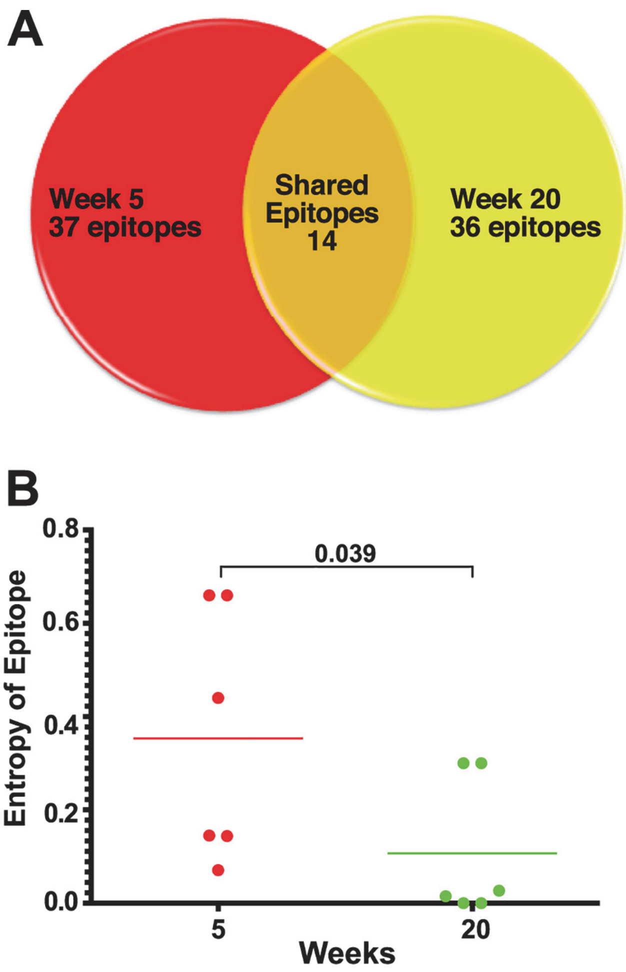

Fig 3. Epitope mapping reveals a change in the CD8 T cell repertoire and in immunodominance. (A) Of the 37 CD8 T cell epitopes mapped for 6 SIVΔnef-vaccinated animals at week 5, only 14 epitopes were recognized at week 20. Approximately two thirds of the epitopes recognized at week 20 were novel responses not previously detected. (B) The entropy of the immunodominant epitope decreased significantly between weeks 5 and 20 (p = .039).

doi:10.1371/journal.ppat.1004633.g003

epitopes. In macaque 225–97, for which we have the most comprehensive set of viral sequence data and longitudinal epitope mapping, almost all of the nonsynonymous mutations (7 out of 9 mutations) with a frequency greater than 50% fell within a mapped CD8 T cell epitope (Fig. 5A), implying a causal link between CD8 T cell pressure and viral escape. Moreover, the nonsynonymous mutations and mapped epitopes not only overlapped spatially but also overlapped temporally. In animals 225–97 and 256–00, epitope-specific CD8 T cell responses declined in frequency following escape of the epitope (Fig. 5B). Remarkably, escape epitopes in animal 225–97 were themselves overtaken by other epitope variants over time, suggesting CD8 T cell pressure on the escape epitopes as well (Fig. 5C).

However, we did not observe sequence variation in all identified CD8 T cell epitopes. As expected, we observed a strong bias favoring the escape of responses targeting highly variable epitopes, as determined by calculated entropy scores. An analysis of the top four responses mapped for each animal at weeks 5, 20 and 40 after SIVΔnef vaccination demonstrated that the escaped epitopes had significantly higher entropy scores than targeted epitopes that did not

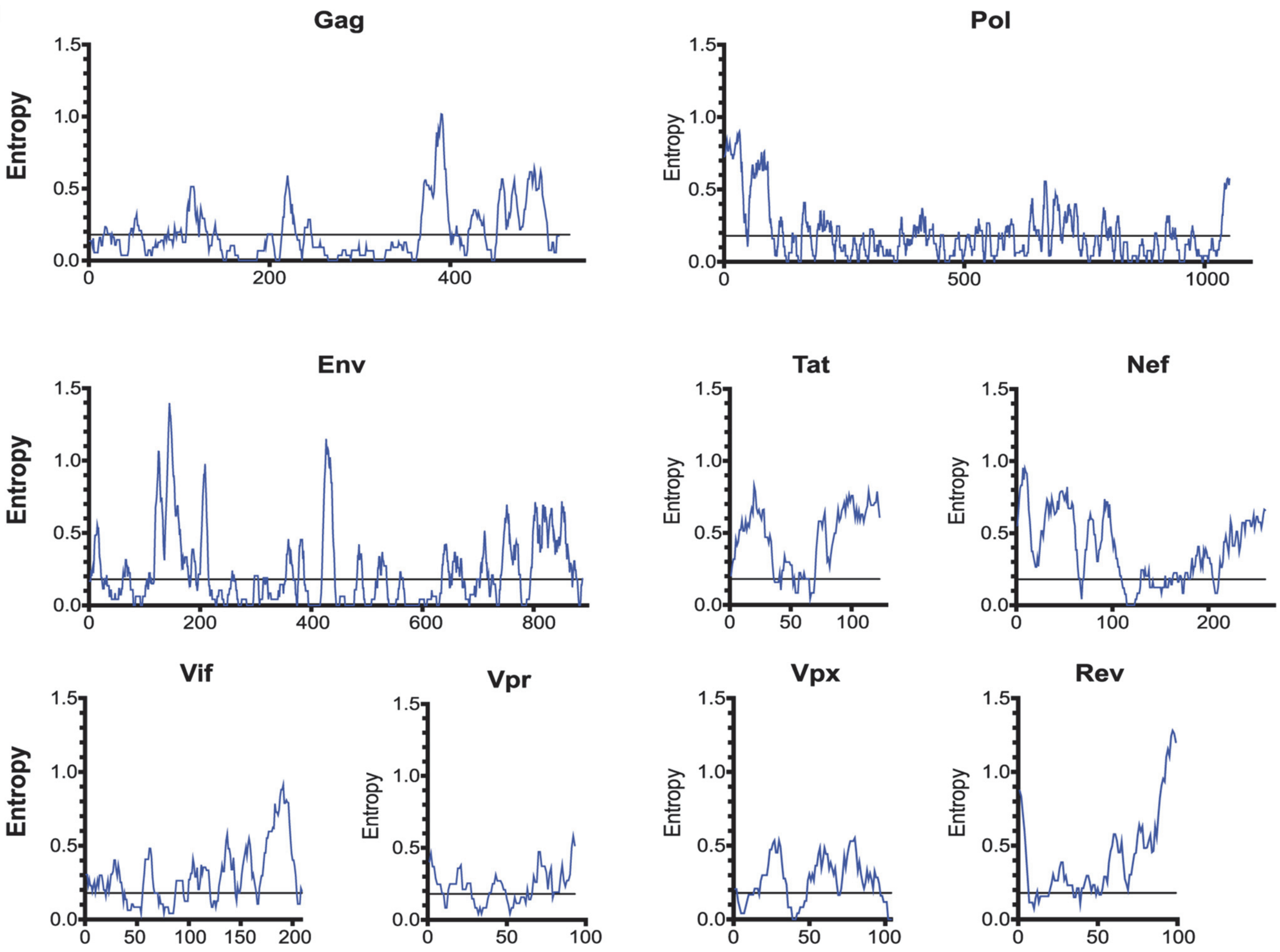

Fig 4. The entropy of SIVsmm proteins varies widely. The entropy of a 9 amino-acid window is graphed at each amino acid position. The entropy of each amino acid was calculated by aligning 10 SIVsmm proteomes.

doi:10.1371/journal.ppat.1004633.g004

escape (p = 0.0013) (Fig. 6A). Similarly, SIV-specific CD8 T cells can be grouped into maintained responses, which target mostly conserved regions, and waning responses that target escaped epitopes. The magnitude of the variable responses, defined as targeting epitopes with an entropy above 0.25, decreased significantly between weeks 5 and 20 (p = .0101) and between weeks 5 and 40 (p = .0449), whereas the magnitude of the conserved responses was maintained (Fig. 6B).

To determine if the selective escape of variable epitopes, coupled with the decline of their CD8 T cell response frequencies, led to an immune response that was increasingly focused on more conserved epitopes, we developed a formula that estimates the conservation level of the immune response. The Response Conservation Index is the sum of all SIV-specific CD8 T cell response frequencies, weighted for entropy. The Response Conservation Index was calculated for every animal at both week 5 and 20 using the formula in Fig. 7. Importantly, in the six animals whose CD8 T cell responses were mapped, the Response Conservation Index increased significantly between weeks 5 and 20 (p = 0.0156) (Fig. 7).

To ascertain that the increased targeting of conserved epitopes in the vaccinated animals was not due to a genetic background enriched for protective MHC I molecules, we conducted comprehensive pyrosequencing of MHC I genotypes in the six animals whose CD8 T cell

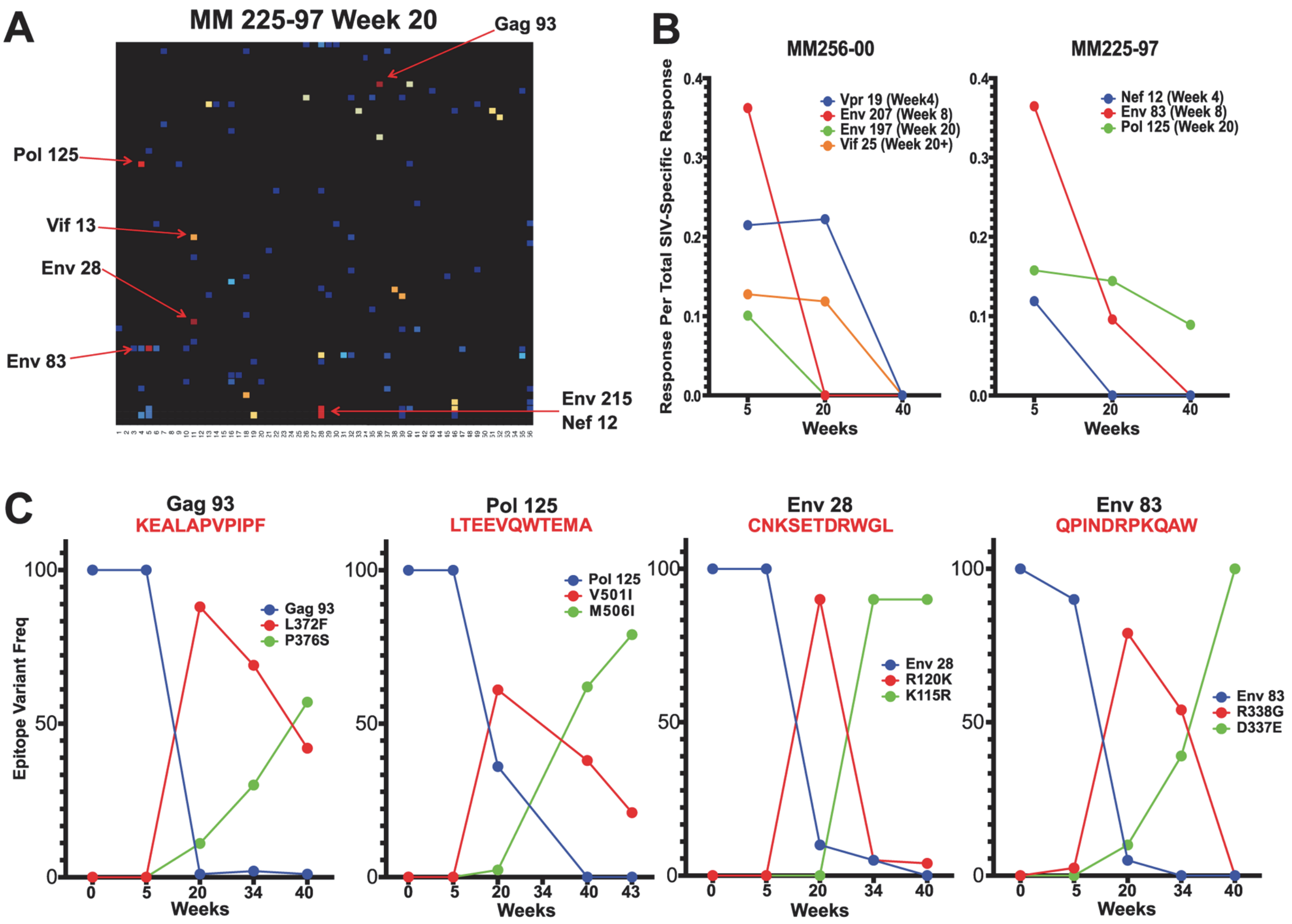

Fig 5. Sequence evolution and CD8 T cell responses overlap, positionally and temporally. (A) In animal 225–97, 7 of the 9 highest frequency nonsynonymous mutations at week 20 were within mapped CD8 T cell epitopes. (B) Epitope-specific CD8 T cell responses declined as their epitopes escaped. Time of 50% escape is shown between parentheses. (C) The sequence evolution of 4 CD8 T cell epitopes mapped from animal 225–97 demonstrates the dynamic nature of viral escape. In all cases, escape variants were over taken by other variants at a later time point.

doi:10.1371/journal.ppat.1004633.g005

responses were longitudinally mapped. The cohort displayed a diverse set of MHC I molecules with three animals expressing the protective alleles Mamu-A∗01 and Mamu-B∗17, while the rest of the animals did not express any protective MHC I alleles (Table 1). Notably, increased anentropic specificity was documented in every animal between weeks 5 and 20 regardless of the MHC I background (Fig. 7).

Epitope escape drives increased breadth and depth of the CD8 T cell response

The number of epitopes targeted by the CD8 T cell response did not differ between weeks 5 and 20, as measured by positive peptide pools (Fig. 2B, 2C) or the specific mapped epitopes (Fig. 8A). However, we hypothesized that escaped CD8 T cell responses were not deleted but rather maintained as a central memory T cell pool and, therefore, that the cumulative breadth of the CD8 T cell repertoire, encompassing all previously detected responses in a given animal, may be a more accurate measure of SIV-specific CD8 T cell breadth (Fig. 8B). Phenotypic analysis of SL8-

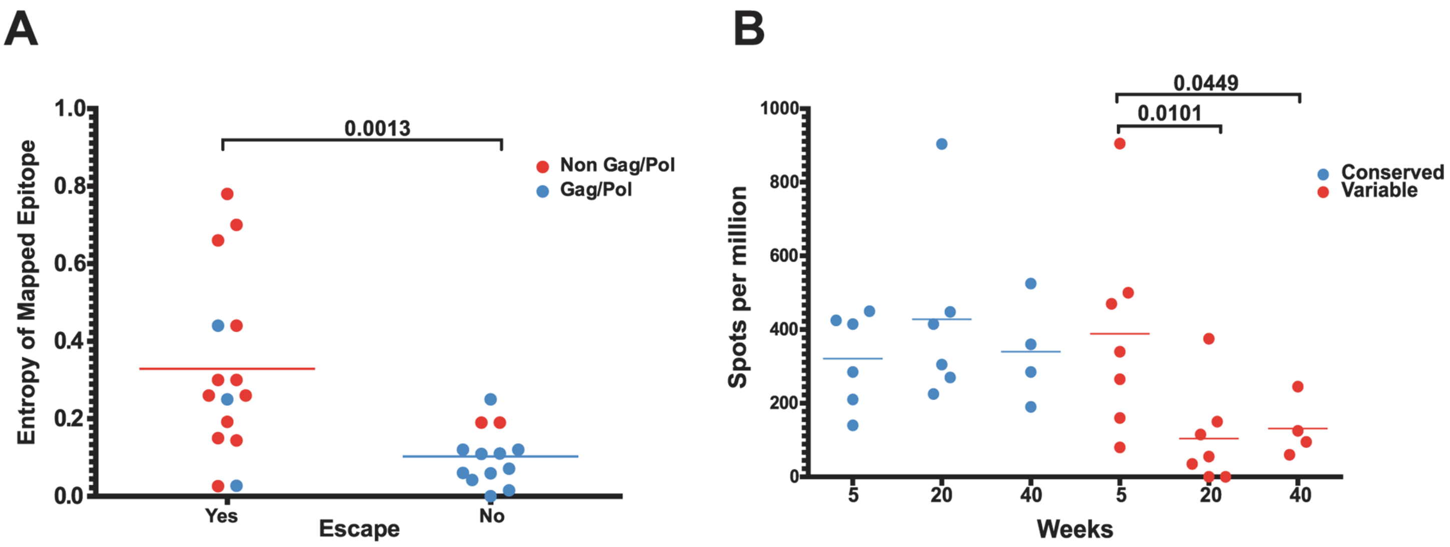

Fig 6. As entropic epitopes escape, the CD8 T cell responses specific for these epitopes wane. (A) The four immunodominant epitopes from all animals were plotted according to their entropies and whether they escaped the CD8 T cell response. Anentropic epitopes demonstrated significantly less escape than highly entropic epitopes. Anentropic epitopes were predominantly from Gag and Pol. (B) While CD8 T cell responses targeting conserved epitopes were maintained over time, responses specific to variable epitopes declined in magnitude.

doi:10.1371/journal.ppat.1004633.g006

specific CD8 T cells, targeting the highly mutable SL8 epitope, did reveal a shift to a central memory profile between weeks 5 and 20, characterized by increased cell surface expression of CD28 ($p = 0.0087$), CCR7 ($p = 0.0006$) and CD127 ($p<0.0001$), as well as a decrease in intracellular expression of perforin ($p<0.0001$) and Ki67 ($p = 0.0194$) (Fig. 8C). In *Mamu-A1∗001+* (previously known as *Mamu-A∗01*) animals, SL8-specific CD8 T cell responses are frequently immunodominant during acute infection but quickly decline following the evolution of escape variants, where

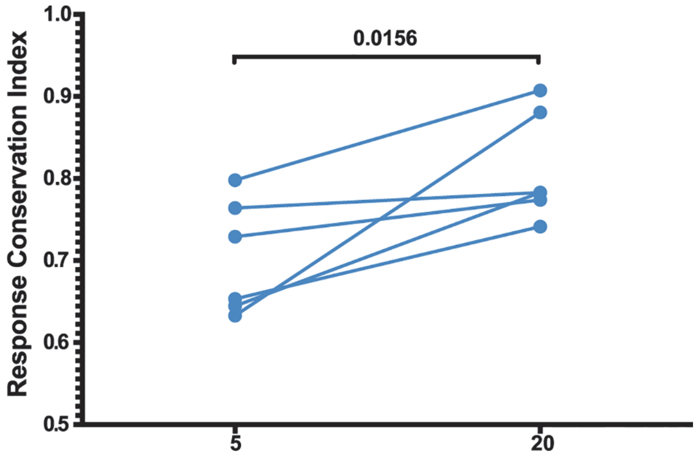

Fig 7. Increased anentropic specificity of SIV-specific CD8 T cell responses between weeks 5 and 20 after SIVΔnef vaccination. The response conservation index, a measure of the CD8 T cell response weighted negatively for entropy, increases for every animal between weeks 5 and 20 ($p = 0.0156$). The response conservation index for an animal at a given time point is calculated as $\sum_{i=0}^{n} F_i(1 - E_i)$, where F is the frequency of each epitope-specific CD8 T cell response as a fraction of the total magnitude of the response, E is the entropy of the epitope, and n is the number of epitope-specific CD8 T cell responses.

doi:10.1371/journal.ppat.1004633.g007

Table 1. Major alleles expressed as determined by MHC I genotyping.

	Animal ID					
	286–07	256–00	211–02	290–07	225–97	292–07
Mamu-A Sequences	*A1*001*	A1*002	A1*004	A1*008	A1*004	*A1*001*
	A1*004	A1*012	A1*025	A1*023	A1*025	
Mamu-B Sequences	B*012	*B*017*	B*005	B*005	B*001	*B*017*
	B*022	B*019	B*040	B*040	B*005	B*029
	B*030	B*024	B*044	B*047	B*007	B*041
	B*031	B*029	B*048		B*030	B*048
	B*043		B*064		B*040	
	B*073				B*044	
	B*074					

Comprehensive MHC I genotyping identified major alleles expressed in the six animals whose CD8 T cell responses were mapped longitudinally. Major MHC I alleles were defined as transcripts constituting at least 4% of total MHC I transcripts for a given animal. MHC I alleles previously correlated with slow SIV disease progression are italicized.

doi:10.1371/journal.ppat.1004633.t001

82% and 94% of sequenced SL8 epitopes had mutated as early as week 4 in two SIVΔnef-vaccinated macaques (Fig. 8D). Although SL8-specific CD8 T cells reached nearly undetectable levels by week 20 as measured by IFN-γ ELISPOT and SL8-MHC tetramer staining, the responses rebounded in both animals following challenge with SIVmac251 (Fig. 8D). Given the maintenance of responses to escaped epitopes in SIVΔnef-vaccinated animals, all escaped responses were included in the calculation of cumulative breadth. In contrast to CD8 T cell breadth, the cumulative breadth of the CD8 T cell response increased significantly from an average of 6 responses per animal at week 5 to an average of 10 responses at week 20 ($p = 0.0036$) (Fig. 8B).

Finally, SIVΔnef escape, in addition to allowing for anentropic specificity and increased cumulative breadth, contributes to the deepening of the CD8 T cell response. In macaque 256–00, the mapped Mamu-B17-restricted FW9 (FHEAVQAVW) epitope was intact at week 5 but the virus fully escaped by week 20 with 99.9% of sequenced reads containing the FW9-H831Y variant. To determine if de novo CD8 T cell responses to the escape variant arose, we tested both variants at week 5 and at the latest time point before challenge, week 38. Lymph node CD8 T cells from macaque 256–00 recognized FW9 at week 5 but not at week 38; conversely, the FW9-H831Y escape variant was recognized only at week 38 but not at week 5 (Fig. 9A). Similarly, the GY9-K76R epitope variant was recognized at week 38 but not at week 5 demonstrates increased depth of the CD8 T cell response after the escape of the mapped Mamu-A∗02-restricted GY9 (GSENLKSLY) epitope (Fig. 9B). These data demonstrate that, at least in a subset of SIVΔnef-vaccinated animals, the depth of the CD8 T cell response increased between weeks 5 and 20 as new CD8 T cell responses were mounted against escape variants.

Discussion

Taken together, this data set supports a comprehensive model that provides new insights into the mechanism of SIV-specific immunity maturation after SIVΔnef vaccination. For the first time, we demonstrate that SIVΔnef sequence evolution is widespread after vaccination, not just confined to the previously documented highly mutable epitope of Tat SL8 [26], and that ongoing sequence evolution occurs even in animals with undetectable plasma viremia. Moreover, we show that the observed sequence evolution can be characterized as viral epitope escape, a

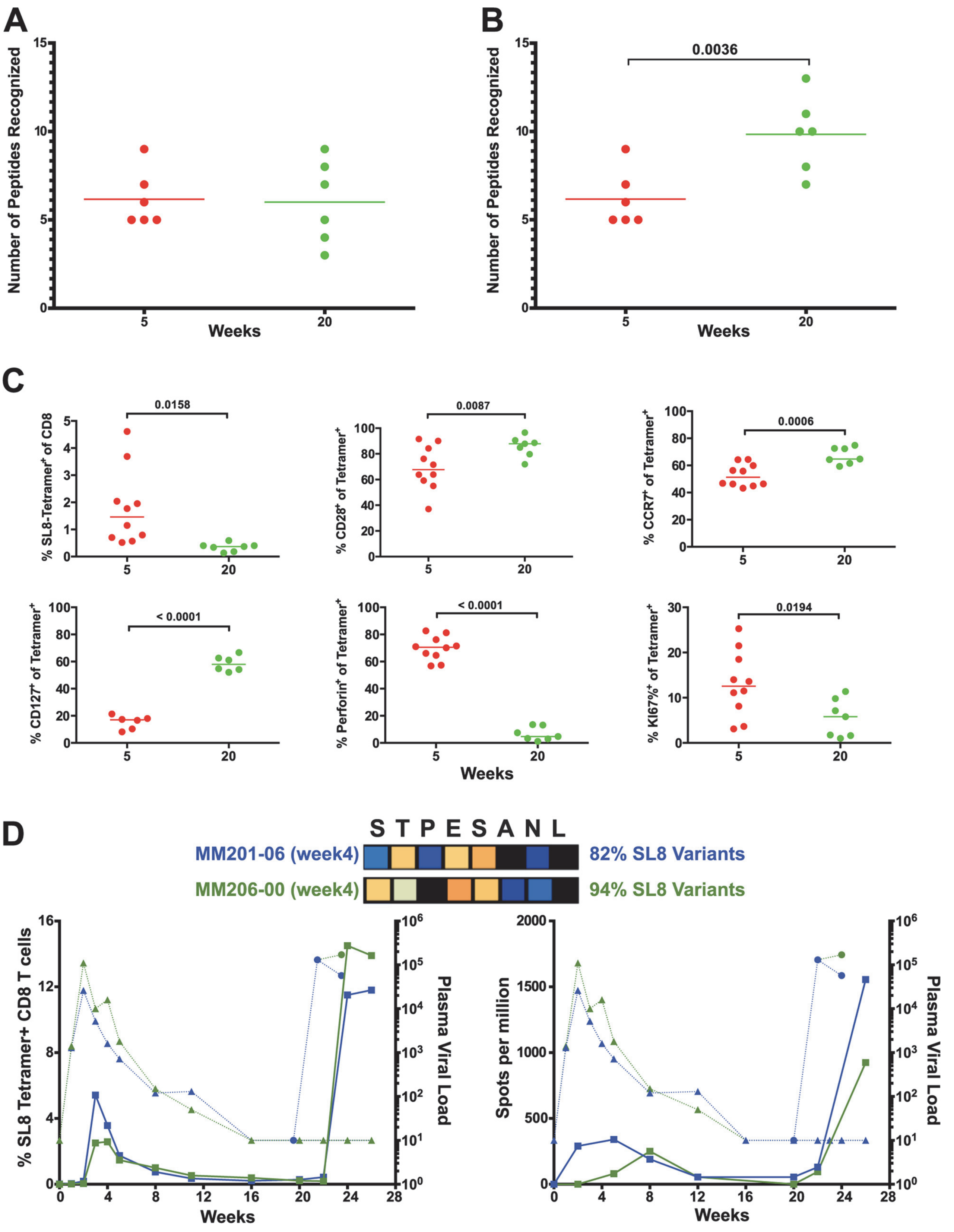
A
Number of Peptides Recognized
Weeks
5
20
B
0.0036
C
0.0158
% SL8-Tetramer+ of CD8
0.0087
% CD28+ of Tetramer+
0.0006
% CCR7+ of Tetramer+
< 0.0001
% CD127+ of Tetramer+
< 0.0001
% Perforin+ of Tetramer+
0.0194
% KI67%+ of Tetramer+
Weeks
D
S T P E S A N L
MM201-06 (week4)
82% SL8 Variants
MM206-00 (week4)
94% SL8 Variants
% SL8 Tetramer+ CD8 T cells
Plasma Viral Load
Spots per million
Weeks

Fig 8. Escaped responses contribute to the cumulative breadth of the CD8 T cell repertoire. (A) No change in breadth of the CD8 T cell response was observed as measured by the number epitope-specific CD8 T cell responses per animal at weeks 5 and 20. (B) The cumulative breadth at week 20 was significantly higher than the breadth at Week 5 (p = 0.0036). The cumulative breadth at week 20 included all the week 5 epitope responses, even if they were not detected at week 20. (C) Phenotypic analysis of SL8 tetramer+ CD8 T cells from peripheral blood. SL8-specific CD8 T cells exhibited a central memory profile at week 20, as demonstrated by increased expression of CD28 (p = 0.0087), CCR7 (p = 0.0006) and CD127 (p<0.0001) markers and a decrease in perforin (p<0.0001) and Ki67 (p = 0.0194). (D) Tat SL8 epitope heat maps showing 82% and 94% percent variation at week 4 for animals 201–02 and 206–00, respectively. Below, two graphs show the primary and recall SL8-specific CD8 T cell responses (squares) as measured by Mamu-A*01-SL8 tetramer staining (left) and IFN-γ ELISPOT (right) in the context of plasma viral loads of SIVΔnef (triangles) and SIVmac251 challenge virus (circles) in 201–02 (dark blue) and 206–00 (green).

doi:10.1371/journal.ppat.1004633.g008

consequence of SIV-specific CD8 T cell pressure on the virus as indicated by the many nonsynonymous mutations that appear within mapped CD8 T cell epitopes. Importantly, another direct consequence of immune CTL pressure is the disproportionate escape of highly variable epitopes, which leads to the waning of CD8 T cell responses targeting entropic epitopes and the accumulation of CD8 T cell responses specific for these epitopes. In addition, escaped SIV-specific CD8 T cell responses are maintained as central memory CD8 T cells and, as such, contribute to the increased cumulative breadth of the CD8 T cell response at week 20 post-vaccination with SIVΔnef. Finally, epitope escape and the ensuing generation of de novo CD8 T cell responses to these escape variants constitute a deepening of the CD8 T cell repertoire.

Our work sheds new light on SIVΔnef and other LASIV vaccines and how their functional and mechanistic characteristics are fundamentally different from other vaccines. While many of the recent successful replication competent virus vector-based vaccines such as CMV and RRV are also persistent [34–37], we demonstrate that SIVΔnef is not only a persistent vaccine but that, as a lentivirus, it evolves with the immune response it induces and, ultimately, shapes the CD8 T cell response during the vaccination period. This unique characteristic of SIVΔnef to mutate allows for maturation of CTL specificity through increased targeting of conserved regions, deepening and broadening of the response. Furthermore, this new model accommodates earlier known properties of LASIV, such as the observation that the level of protection of LASIV vaccines inversely correlates with the level of attenuation of the vaccine [6,38]. Based on our current work, we hypothesize that LASIV vaccine efficacy depends on virus evolution, resulting in the concurrent CD8 T cell specificity shift from highly variable to conserved regions of the virus. Highly attenuated viruses, such as SIVΔ4, which exhibits lower efficacy compared

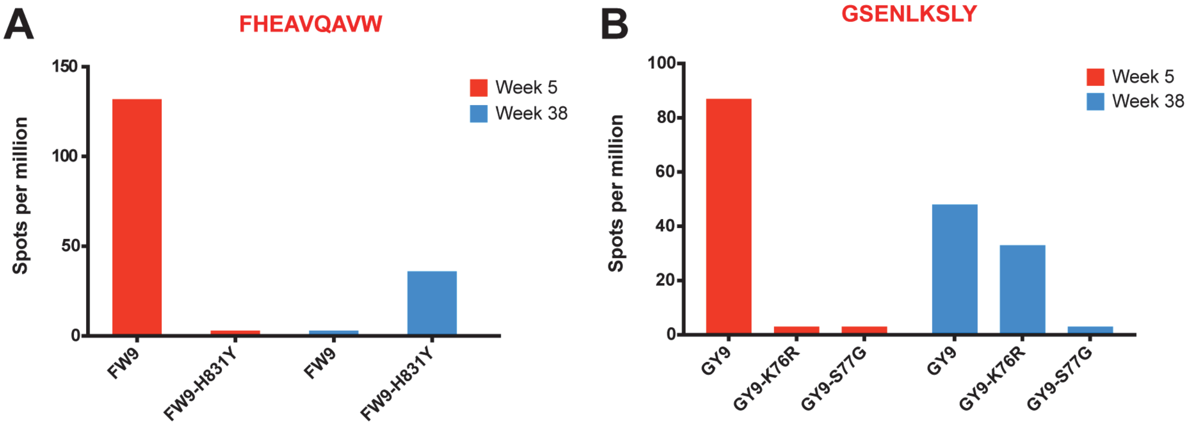

Fig 9. Increased CD8 T cell repertoire depth during SIVΔnef vaccination. (A) FW9, an epitope mapped in animal 256–00, escaped the CD8 T cell response by mutating a histidine into a tyrosine (H831Y). The escape mutant was recognized at week 38 but not at week 5, when the original epitope was mapped. (B) The GY9 epitope was recognized at week 5 in animal 256–00 as well as week 38, while its escape variant K76R was recognized only at week 38. The third escape variant S77G was not recognized at either time point.

doi:10.1371/journal.ppat.1004633.g009

to SIVΔnef [38], would have lower replication rates and less escape events, therefore less opportunity to shape the CD8 T cell response.

Despite three decades of HIV research, basic questions of vaccine design remain hotly debated, namely the optimal balance of CD8 T cell response magnitude, breadth and specificity that should be induced by a vaccine. In suggesting that anentropic specificity is a more important correlate of protection in SIVΔnef-vaccinated animals than response magnitude, our data corroborates previous work on long-term nonprogressors (LTNP), another important model of spontaneous lentiviral suppression. Research on LTNP has shown that MHC I molecules associated with protection restrict CD8 T cells that target regions within HIV Gag [39,40] as well as highly conserved epitopes [41]. However, unlike viral control in LTNP, which is associated with certain protective HLA molecules, we document increased targeting of highly conserved epitopes in all animals regardless of their MHC I genotypic background. Similar to our LASIV-induced protection, viral control in LTNP is correlated with the immunodominance of CD8 T cell responses that target highly conserved epitopes [42], given that immunodominant CD8 T cells exert more immune pressure [41]. Finally, studies of LTNP revealed the importance of targeting conserved regions during acute infection [41], before massive depletion of CD4 T cells has occurred [43]. Our research demonstrates that at week 20, whereas responses to variable regions have waned, the immunodominant SIV-specific CD8 T cells target more conserved epitopes than at week 5. The presence of immunodominant CD8 T cells exerting pressure on highly conserved regions during acute infection in animals challenged at week 20 post-vaccination, but not at week 5, could explain the observed difference in protection. Conversely, the absence of immunodominant responses targeting highly conserved regions in animals challenged at week 5 allows the virus to easily escape and the accumulation of anentropic-specific CD8 T cell responses would occur long after acute infection, if at all.

In addition to immunodominant SIV-specific CD8 T cells with anentropic specificity, we also show increased cumulative breadth and depth of the CD8 T cell response at week 20 compared to week 5. The increased cumulative breadth at week 20 is a function of waning escaped CD8 T cell responses that are maintained as central memory CD8 T cells and are recalled upon challenge. It is unclear how effective these responses are at long-term viral suppression, given that they have been escaped and mostly target variable regions of the virus. Still, a recent report suggests that acute-phase SIV-specific CD8 T cells targeting variable regions are important for viral control [44], indicating that the increased cumulative breadth of the CD8 T cell response at week 20 may also contribute to protection. Moreover, we detected de novo CD8 T cell responses specific to escaped epitope variants, illustrating that viral escape also contributes to increased immune response depth. Although LTNP generate HIV-specific CD8 T cells that cross-react with escape mutants [45], the generation of de novo CD8 T cell responses to escaped epitopes is rarely observed in adults infected with HIV [46]. In animal 256–00, we identified two such CD8 T cell responses that target variant epitopes at week 38 but were not detectable at week 5. We only assayed CD8 T cell responses against escape variants of known CD8 T cell epitopes with known MHC I restriction, and only if the MHC I is expressed by the infected animal. The relative frequency of de novo CD8 T cell responses in SIVΔnef-vaccinated animals, if verified in future studies, could be due to an intact CD4 T cell compartment that persists well into the chronic phase of LASIV infection.

Previous reports have documented rapid escape of variable epitopes during acute HIV-1 infection and maintenance of CD8 T cell responses specific for conserved epitopes into chronic infection [47–49]. Here, in the context of SIVΔnef, we demonstrate, in addition to rapid escape of variable epitopes and maintenance of existing CD8 T cell responses with anentropic specificity, *de novo* CD8 T cell responses against novel epitopes and escape variants during chronic infection. Furthermore, we correlate the accumulation of CD8 T cell responses with anentropic

specificity and the increased repertoire depth and breadth to the kinetics of SIVΔnef vaccine-induced protection. Previous research on the coevolution of lentiviral epitopes and the CD8 T cell response has been conducted in the setting of wild-type infection, and it is unclear that our findings of increased anentropic specificity, cumulative breadth and repertoire depth during SIVΔnef vaccination would apply to wild-type lentiviral infection, in which the CD8 T cell response is likely to be impaired by the persistently high antigenic load, CD4 T cell depletion in the gut and microbial translocation-induced immune activation.

Notably, we did not see an increased magnitude of the SIV-specific CD8 T cell response in the lymph nodes between weeks 5 and 20. Fukazawa and colleagues [6] have recently shown that less attenuated LASIVs such as SIVΔnef have higher viral replication in the lymph nodes and, in turn, have higher lymph node CD8 T cell responses. Although these results corroborate existing data demonstrating that protection inversely correlates with LASIV attenuation [38], our data demonstrates that the magnitude of the CD8 T cell response does not correlate with the kinetics of protection, neither in the PBMC nor lymph node compartment. Although our current work supports the importance of conserved responses as a correlate for protection, others have shown that using conserved regions as immunogens may result in a reduction of the total magnitude of responses [50]. It is unknown whether vaccines inducing targeted responses toward conserved regions will make up for the loss of magnitude. Still it is encouraging that we observe increased anentropic specificity in all animals, suggesting that even nonprotective MHC I molecules can target highly conserved epitopes if properly primed.

It has also been proposed that the increased protection of LASIV vaccines is due to phenotypic maturation of the CD8 T cell response. It is important to delineate whether observed phenotypic changes in SIV-specific CD8 T cells correlate with LASIV protection and whether CD8 T cell phenotypic maturation is specific to LASIV or can be seen in other vaccines, such as adenovirus-, CMV- and RRV-based SIV vaccines. Finally, although this data set was focused on the CD8 T cell response and its evolution over time post-vaccination, we observed relatively few nonsynonymous mutations in *env* outside of mapped CD8 T cell epitopes, suggesting that antibody responses induced only modest immune selective pressure on SIVΔnef compared to CD8 T cell responses.

Although immunodominance, specificity, breadth and depth have been implicated for some time as determinants of a successful CD8 T cell response against HIV/SIV infection, these results demonstrate how such a CD8 T cell response may be induced by the highly protective SIVΔnef vaccine. This new model of SIVΔnef-mediated protection raises obvious implications for vaccine design, namely that a successful vaccine should focus CD8 T cell responses on conserved regions of the virus and their existing variants. The recent success of viral vectors such as CMV [34–36], RRV [37], and adenovirus [51] to elicit potent CD8 T cell responses that partially protect rhesus macaques from infection has been an important step forward in vaccine design. However, these vaccines are all derived from DNA viruses, which have significantly lower rates of mutation than lentiviruses, and thus they are unlikely to shape the immune response in a similar fashion to SIVΔnef. Our work suggests that immunogens should be carefully chosen for these vectors such that the induced responses preferentially target conserved regions of the virus, as well as existing variants of these regions to increase response breadth and depth simultaneously.

Supporting Information

S1 Table. Epitope mapping of the CD8 T cell response in peripheral blood and lymph nodes. The mapped CD8 T cell response in peripheral blood (PBMC, left) and lymph nodes (LN, right) for animal 225–97 at week 40 and animal 211–02 at week 20 post-SIVΔnef

vaccination. The frequency of an epitope-specific response per 10^6 cells and the epitope sequence are listed. Concordant CD8 T cell responses between peripheral blood and lymph nodes are shaded in green.
(DOCX)

S1 Fig. Sequence variation at week 2 is characterized by low frequency mutations. Heat maps of SIVΔnef sequence evolution at week 2 in animals 376–02, 256–00 and 286–07. The top left corner is the first amino acid of Gag and the bottom right corner is the last expressed amino acid of the truncated Nef.
(EPS)

S2 Fig. At week 4, early purifying selection of nonsynonymous mutations was observed in SIVΔnef-vaccinated animals. Heat maps of SIVΔnef sequence evolution at week 4 in 6 vaccinated animals. By week 4, there were 6 to 12 nonsynonymous mutations with a frequency greater than 10% per animal. The top left corner is the first amino acid of Gag and the bottom right corner is the last expressed amino acid of the truncated Nef.
(EPS)

S3 Fig. Extensive sequence evolution of SIVΔnef at week 20. Heat maps of SIVΔnef sequence evolution at week 20 in 256–00 and 376–02. By week 20, there was an average of 28 nonsynonymous mutations with a frequency greater than 10% in sequenced plasma virus. The top left corner is the first amino acid of Gag and the bottom right corner is the last expressed amino acid of the truncated Nef.
(EPS)

S4 Fig. The epitope-specific CD8 T cell repertoire changed dramatically between week 5 and 20. Mapped CD8 T cell epitopes for 6 SIVΔnef-vaccinated animals at weeks 5 (red), week 20 (yellow) and epitopes that are mapped at both time points (orange). The top left corner is the first amino acid of Gag and the bottom right corner is the last expressed amino acid of the truncated Nef.
(EPS)

Acknowledgments

The authors would like to dedicate this paper to the memory of Michael Piatak, whose experimental rigor, dedication, and humanity inspired all of us. We thank David O'Connor and Roger Wiseman for MHC I genotyping, Ronald Desrosiers for providing SIVΔnef, Yuan Li for assistance with quantitative determinations of SIV viral loads, Angela Carville and members of the NEPRC Primate Medicine staff for expert animal care, and Carolyn O'Toole and Noel Bane for expert manuscript preparation.

Author Contributions

Conceived and designed the experiments: RPJ SA. Performed the experiments: SA YY JG FEW EAB MP RKR. Analyzed the data: SA RPJ JDL ADC SLO RKR. Wrote the paper: SA RPJ.

References

1. Daniel MD, Kirchhoff F, Czajak SC, Sehgal PK, Desrosiers RC (1992) Protective effects of a live attenuated SIV vaccine with a deletion in the nef gene. Science 258: 1938–1941. PMID: 1470917
2. Connor RI, Montefiori DC, Binley JM, Moore JP, Bonhoeffer S, et al. (1998) Temporal analyses of virus replication, immune responses, and efficacy in rhesus macaques immunized with a live, attenuated simian immunodeficiency virus vaccine. J Virol 72: 7501–7509. PMID: 9696847

3. Tenner-Racz K, Stahl Hennig C, Uberla K, Stoiber H, Ignatius R, et al. (2004) Early protection against pathogenic virus infection at a mucosal challenge site after vaccination with attenuated simian immunodeficiency virus. Proc Natl Acad Sci USA 101: 3017–3022. PMID: 14970317
4. Reynolds MR, Weiler AM, Piaskowski SM, Kolar HL, Hessell AJ, et al. (2010) Macaques vaccinated with simian immunodeficiency virus SIVmac239Δnef delay acquisition and control replication after repeated low-dose heterologous SIV challenge. J Virol 84: 9190–9199. doi: 10.1128/JVI.00041-10 PMID: 20592091
5. Reynolds MR, Weiler AM, Weisgrau KL, Piaskowski SM, Furlott JR, et al. (2008) Macaques vaccinated with live-attenuated SIV control replication of heterologous virus. J Exp Med 205: 2537–2550. doi: 10.1084/jem.20081524 PMID: 18838548
6. Fukazawa Y, Park H, Cameron MJ, Lefebvre F, Lum R, et al. (2012) Lymph node T cell responses predict the efficacy of live attenuated SIV vaccines. Nat Med 18: 1673–1681. doi: 10.1038/nm.2934 PMID: 22961108
7. Veazey RS, DeMaria M, Chalifoux LV, Shvetz D, Pauley D, et al. (1998) The gastrointestinal tract as a major site of CD4 T lymphocyte depletion and viral replication in SIV infection. Science 280: 427–431. PMID: 9545219
8. Baba TW, Jeong YS, Pennick D, Bronson R, Greene MF, et al. (1995) Pathogenicity of live, attenuated SIV after mucosal infection of neonatal macaques. Science 267: 1820–1825. PMID: 7892606
9. Wyand MS, Manson KH, Lackner AA, Desrosiers RC (1997) Resistance of neonatal monkeys to live attenuated vaccine strains of simian immunodeficiency virus. Nat Med 3: 32–36. PMID: 8986737
10. Baba TW, Liska V, Khimani AH, Ray NB, Dailey PJ, et al. (1999) Live attenuated, multiply deleted simian immunodeficiency virus causes AIDS in infant and adult macaques. Nat Med 5: 194–203. PMID: 9930868
11. Desrosiers RC, Lifson JD, Gibbs JS, Czajak SC, Howe AM, et al. (1998) Identification of highly attenuated mutants of simian immunodeficiency virus. J Virol 72: 1431–1437. PMID: 9445045
12. Alpert MD, Harvey JD, Lauer WA, Reeves RK, Piatak M Jr., et al. (2012) ADCC develops over time during persistent infection with live-attenuated SIV and is associated with complete protection against SIV (mac)251 challenge. PLoS Pathog 8: e1002890. doi: 10.1371/journal.ppat.1002890 PMID: 22927823
13. Schmitz JE, Johnson RP, McClure HM, Manson KH, Wyand MS, et al. (2005) Effect of CD8+ lymphocyte depletion on virus containment after simian immunodeficiency virus SIVmac251 challenge of live attenuated SIVmac239delta3-vaccinated rhesus macaques. J Virol 79: 8131–8141. PMID: 15956558
14. Johnson RP, Glickman RL, Yang JQ, Kaur A, Dion JT, et al. (1997) Induction of vigorous cytotoxic T-lymphocyte responses by live attenuated simian immunodeficiency virus. J Virol 71: 7711–7718. PMID: 9311855
15. Nixon DF, Donahoe SM, Kakimoto WM, Samuel RV, Metzner KJ, et al. (2000) Simian immunodeficiency virus-specific cytotoxic T lymphocytes and protection against challenge in rhesus macaques immunized with a live attenuated simian immunodeficiency virus vaccine. Virology 266: 203–210. PMID: 10612675
16. Genesca M, Skinner PJ, Bost KM, Lu D, Wang Y, et al. (2008) Protective attenuated lentivirus immunization induces SIV-specific T cells in the genital tract of rhesus monkeys. Mucosal Immunol 1: 219–228. doi: 10.1038/mi.2008.6 PMID: 19079181
17. Walker CM, Moody DJ, Stites DP, Levy JA (1986) CD8+ lymphocytes can control HIV replication in vitro by suppressing virus replication. Science 234: 1563–1566. PMID: 2431484
18. Collins KL, Chen BK, Kalams SA, Walker BD, Baltimore D (1998) HIV-1 Nef protein protects infected primary cells against killing by cytotoxic T lymphocytes. Nature 391: 397–401. PMID: 9450757
19. Yang OO, Sarkis PT, Trocha A, Kalams SA, Johnson RP, et al. (2003) Impacts of avidity and specificity on the antiviral efficiency of HIV-1-specific CTL. J Immunol 171: 3718–3724. PMID: 14500671
20. Gauduin M-C, Glickman RL, Means R, Johnson RP (1998) Inhibition of simian immunodeficiency virus (SIV) replication by CD8+ T lymphocytes from macaques immunized with live attenuated SIV. J Virol 72: 6315–6324. PMID: 9658070
21. Tsubota H, Lord CI, Watkins DI, Morimoto C, Letvin N (1989) A cytotoxic T lymphocyte inhibits acquired immunodeficiency syndrome virus replication in peripheral blood lymphocytes. J Exp Med 169: 1421–1434. PMID: 2784486
22. Jin X, Bauer DE, Tuttleton SE, Lewin S, Gettie A, et al. (1999) Dramatic rise in plasma viremia after CD8(+) T cell depletion in simian immunodeficiency virus-infected macaques. J Exp Med 189: 991–998. PMID: 10075982
23. Genesca M, Skinner PJ, Hong JJ, Li J, Lu D, et al. (2008) With minimal systemic T-cell expansion, CD8+ T Cells mediate protection of rhesus macaques immunized with attenuated simian-human

immunodeficiency virus SHIV89.6 from vaginal challenge with simian immunodeficiency virus. J Virol 82: 11181–11196. doi: 10.1128/JVI.01433-08 PMID: 18787003

24. Manrique J, Piatak M, Lauer W, Johnson W, Mansfield K, et al. (2013) Influence of mismatch of Env sequences on vaccine protection by live attenuated simian immunodeficiency virus. J Virol 87: 7246–7254. doi: 10.1128/JVI.00798-13 PMID: 23637396
25. Wyand MS, Manson KH, Garcia-Moll M, Montefiori D, Desrosiers RC (1996) Vaccine protection by a triple deletion mutant of simian immunodeficiency virus. J Virol 70: 3724–3733. PMID: 8648707
26. Burwitz BJ, Ende Z, Sudolcan B, Reynolds MR, Greene JM, et al. (2011) Simian Immunodeficiency Virus SIVmac239{Delta}nef Vaccination Elicits Different Tat28-35SL8-Specific CD8+ T-Cell Clonotypes Compared to a DNA Prime/Adenovirus Type 5 Boost Regimen in Rhesus Macaques. J Virol 85: 3683–3689. doi: 10.1128/JVI.02112-10 PMID: 21270159
27. National Research Council of the National Academies. (2011) Guide for the Care and use of Laboratory Animals. National Institutes of Health. National Academies Press. doi: 10.1080/17437199.2011.587961 PMID: 25473706
28. Salisch NC, Kaufmann DE, Awad AS, Reeves RK, Tighe DP, et al. (2010) Inhibitory TCR coreceptor PD-1 is a sensitive indicator of low-level replication of SIV and HIV-1. J Immunol 184: 476–487. doi: 10.4049/jimmunol.0902781 PMID: 19949078
29. Wiseman RW, Karl JA, Bimber BN, O'Leary CE, Lank SM, et al. (2009) Major histocompatibility complex genotyping with massively parallel pyrosequencing. Nat Med 15: 1322–1326. doi: 10.1038/nm.2038 PMID: 19820716
30. Macchia I, Gauduin MC, Kaur A, Johnson RP (2006) Expression of CD8alpha identifies a distinct subset of effector memory CD4+ T lymphocytes. Immunology 119: 232–242. PMID: 16836648
31. Jia B, Ng SK, DeGottardi MQ, Piatak M, Yuste E, et al. (2009) Immunization with single-cycle SIV significantly reduces viral loads after an intravenous challenge with SIV(mac)239. PLoS Pathog 5: e1000272. doi: 10.1371/journal.ppat.1000272 PMID: 19165322
32. Roederer M (2001) Spectral compensation for flow cytometry: visualization artifacts, limitations, and caveats. Cytometry 45: 194–205. PMID: 11746088
33. Sugimoto C, Tadakuma K, Otani I, Moritoyo T, Akari H, et al. (2003) nef gene is required for robust productive infection by simian immunodeficiency virus of T-cell-rich paracortex in lymph nodes. J Virol 77: 4169–4180. PMID: 12634375
34. Hansen SG, Ford JC, Lewis MS, Ventura AB, Hughes CM, et al. (2011) Profound early control of highly pathogenic SIV by an effector memory T-cell vaccine. Nature 473: 523–527. doi: 10.1038/nature10003 PMID: 21562493
35. Hansen SG, Piatak M Jr., Ventura AB, Hughes CM, Gilbride RM, et al. (2013) Immune clearance of highly pathogenic SIV infection. Nature 502: 100–104. doi: 10.1038/nature12519 PMID: 24025770
36. Hansen SG, Sacha JB, Hughes CM, Ford JC, Burwitz BJ, et al. (2013) Cytomegalovirus vectors violate CD8+ T cell epitope recognition paradigms. Science 340: 1237874. doi: 10.1126/science.1237874 PMID: 23704576
37. Bilello JP, Manrique JM, Shin YC, Lauer W, Li W, et al. (2011) Vaccine Protection against Simian Immunodeficiency Virus in Monkeys Using Recombinant Gamma-2 Herpesvirus. J Virol 85: 12708–12720. doi: 10.1128/JVI.00865-11 PMID: 21900170
38. Johnson RP, Lifson JD, Czajak SC, Cole KS, Manson KH, et al. (1999) Highly attenuated vaccine strains of simian immunodeficiency virus protect against vaginal challenge: inverse relationship of degree of protection with level of attenuation. J Virol 73: 4952–4961. PMID: 10233957
39. Kiepiela P, Ngumbela K, Thobakgale C, Ramduth D, Honeyborne I, et al. (2007) CD8+ T-cell responses to different HIV proteins have discordant associations with viral load. Nat Med 13: 46–53. PMID: 17173051
40. Streeck H, Lichterfeld M, Alter G, Meier A, Teigen N, et al. (2007) Recognition of a defined region within p24 gag by CD8+ T cells during primary human immunodeficiency virus type 1 infection in individuals expressing protective HLA class I alleles. J Virol 81: 7725–7731. PMID: 17494064
41. Wang YE, Li B, Carlson JM, Streeck H, Gladden AD, et al. (2009) Protective HLA class I alleles that restrict acute-phase CD8+ T-cell responses are associated with viral escape mutations located in highly conserved regions of human immunodeficiency virus type 1. J Virol 83: 1845–1855. doi: 10.1128/JVI.01061-08 PMID: 19036810
42. Liu MK, Hawkins N, Ritchie AJ, Ganusov VV, Whale V, et al. (2013) Vertical T cell immunodominance and epitope entropy determine HIV-1 escape. J Clin Invest 123: 380–393. doi: 10.1172/JCI65330 PMID: 23221345

43. Mattapallil JJ, Douek DC, Hill BJ, Nishimura Y, Martin M, et al. (2005) Massive infection and loss of memory CD4+ T cells in multiple tissues during acute SIV infection. Nature 434: 1093–1097. PMID: 15793563

44. Harris M, Burns CM, Becker EA, Braasch AT, Gostick E, et al. (2013) Acute-phase CD8 T cell responses that select for escape variants are needed to control live attenuated simian immunodeficiency virus. J Virol 87: 9353–9364. doi: 10.1128/JVI.00909-13 PMID: 23785211

45. Chen H, Ndhlovu ZM, Liu D, Porter LC, Fang JW, et al. (2012) TCR clonotypes modulate the protective effect of HLA class I molecules in HIV-1 infection. Nat Immunol 13: 691–700. doi: 10.1038/ni.2342 PMID: 22683743

46. Feeney ME, Tang Y, Pfafferott K, Roosevelt KA, Draenert R, et al. (2005) HIV-1 viral escape in infancy followed by emergence of a variant-specific CTL response. J Immunol 174: 7524–7530. PMID: 15944251

47. Ganusov VV, Goonetilleke N, Liu MK, Ferrari G, Shaw GM, et al. (2011) Fitness costs and diversity of the cytotoxic T lymphocyte (CTL) response determine the rate of CTL escape during acute and chronic phases of HIV infection. J Virol 85: 10518–10528. doi: 10.1128/JVI.00655-11 PMID: 21835793

48. Liu Y, McNevin JP, Holte S, McElrath MJ, Mullins JI (2011) Dynamics of viral evolution and CTL responses in HIV-1 infection. PLoS One 6: e15639. doi: 10.1371/journal.pone.0015639 PMID: 21283794

49. Yang OO, Daar ES, Ng HL, Shih R, Jamieson BD (2011) Increasing CTL targeting of conserved sequences during early HIV-1 infection is correlated to decreasing viremia. AIDS Res Hum Retroviruses 27: 391–398. doi: 10.1089/aid.2010.0183 PMID: 21087140

50. Stephenson KE, SanMiguel A, Simmons NL, Smith K, Lewis MG, et al. (2012) Full-length HIV-1 immunogens induce greater magnitude and comparable breadth of T lymphocyte responses to conserved HIV-1 regions compared with conserved-region-only HIV-1 immunogens in rhesus monkeys. J Virol 86: 11434–11440. doi: 10.1128/JVI.01779-12 PMID: 22896617

51. Barouch DH, Liu J, Li H, Maxfield LF, Abbink P, et al. (2012) Vaccine protection against acquisition of neutralization-resistant SIV challenges in rhesus monkeys. Nature 482: 89–93. doi: 10.1038/nature10766 PMID: 22217938
